# Impaired neural mechanism for online novel word acquisition in dyslexic children

**DOI:** 10.1038/s41598-018-31211-0

**Published:** 2018-08-24

**Authors:** Lilli Kimppa, Yury Shtyrov, Eino Partanen, Teija Kujala

**Affiliations:** 1 Cognitive Brain Research Unit, Department of Psychology and Logopedics, Faculty of Medicine, University of Helsinki, Helsinki, Finland; 2 Center of Functionally Integrative Neuroscience, Department of Clinical Medicine, Aarhus University, Aarhus, Denmark; 3 St. Petersburg State University, St. Petersburg, Russian Federation

## Abstract

Developmental dyslexia is characterised as an inability to read fluently. Apart from literacy problems, dyslexics have other language difficulties including inefficient speech encoding and deficient novel word learning. Yet, the neural mechanisms underlying these impairments are largely unknown. We tracked online formation of neural memory traces for a novel spoken word-form in dyslexic and normal-reading children by recording the brain’s electrophysiological response dynamics in a passive perceptual exposure session. Crucially, no meaning was assigned to the new word-form nor was there any task related to the stimulus, enabling us to explore the memory-trace formation of a purely phonological form in the absence of any short-term or working memory demands. Similar to previously established neural index of rapid word learning in adults, the control children demonstrated an early brain response enhancement within minutes of exposure to the novel word-form that originated in frontal cortices. Dyslexic children, however, lacked this neural enhancement over the entire course of exposure. Furthermore, the magnitude of the rapid neural enhancement for the novel word-form was positively associated with reading and writing fluency. This suggests that the rapid neural learning mechanism for online acquisition of novel speech material is associated with reading skills. Furthermore, the deficient online learning of novel words in dyslexia, consistent with poor rapid adaptation to familiar stimuli, may underlie the difficulty of learning to read.

## Introduction

Developmental dyslexia, a specific reading impairment accompanied by compromised spelling and writing, is one of the most prevalent learning disorders, affecting 5–17% of children with normal IQ and educational possibilities^1,2^. Deficit in phonological processing is widely considered as the underlying cause of dyslexia^3,4^. According to one view, phonological forms (such as words) are poorly constructed in dyslexia; they are inaccurate and incomplete in their segmental organisation^5,6^, implying inefficient memory encoding in the first place. Underdeveloped phonological word-forms may hinder successful phonology-to-orthography mapping that is necessary in reading and writing. Yet, the neural mechanisms behind learning of novel linguistic representations in dyslexia are not understood.

Word learning studies in dyslexia primarily employ acquisition of a novel word-form (merely the sound of the word) in association with another, already familiar word or with a visual referent which provide the meaning of the new word. It is, however, imperative to disentangle these two factors with respect to their contribution into the learning deficit. The evidence so far indicates a learning impairment specific to novel phonological forms, with fluent acquisition of semantic and visual associations^7–14^. These studies could not establish, however, whether it is specifically the stage of initial memory encoding, retrieval, or production of the newly learnt word that produces the difficulty. Some evidence suggests that the origin of impaired learning in dyslexia stems from the perceptual encoding level^15^. This account postulates that dyslexics do not benefit from stimulus-specific repetition, unlike normal readers who form “a perceptual anchor” and automatically link subsequent repetitions to it^16–18^ (note that somewhat similar phonological deficits have been found in SLI^19^ although it is important to differentiate SLI from dyslexia). In similar vein, findings of impaired implicit learning of both novel auditory categories^20^ and motor sequences in dyslexia^21,22^ corroborate the notion that repetition does not facilitate learning in dyslexia as much as it does in normally reading individuals. In the context of word learning, it remains unknown whether it is particularly the failure to benefit from repetitions at the perceptual level (found for both linguistic and non-linguistic stimuli) that slows down the word encoding process, or whether other neural processes underlie the difficulty.

At the neural level, no electrophysiological studies have been conducted on word acquisition in dyslexia. Recent evidence indicates a fundamentally reduced neural adaptation to a variety of verbal and non-verbal stimuli in dyslexic children and adults^23^. Neural adaptation refers to suppression of the neurophysiological response to a stimulus when it is repeated^24^, and is suggested to be the mechanism underlying perceptual learning and memory-trace formation^25–27^. The diminished response suppression in dyslexics compared to normally reading controls has so far been observed with the repetition of familiar words, i.e. the dysfunctional adaptation was not related to learning of new words. Reduced adaptation of cortical response to familiar words may indicate impaired short-term memory processes, but it is not evident whether this impairment impacts normal rapid memory-trace formation for *novel* stimuli.

To test this experimentally, we employed brief repetitive exposure to a new word-form, i.e. a phonotactically plausible sequence of familiar native phonemes combined in a novel way that formed a pseudo-word with no meaning. To quantify the neural processes of new word-form acquisition during the session of repetitive passive exposure, we recorded online neural activity with EEG in both dyslexic children and matched controls. Recently, a robust neural index of rapid memory-trace formation for novel word-forms during brief (<15 min) exposure was discovered in adults and children with passive paradigms using single or multiple novel word tokens^28–31^. The memory-trace formation is reflected as an amplitude increase of the early (<100 ms after word disambiguation point) event-related potential (ERP) to novel spoken forms only, whereas responses to known words tend to attenuate due to repetition. Crucially, this dynamics correlates with behavioural word learning outcomes and manifests similarly well in passive perceptual exposure and in attentive listening conditions, suggesting that it reflects the initial automatic neural process involved in word-form acquisition^30^.

To exclude any excessive demands on short-term memory and attention, no task or focused attention on the stimuli were involved in this study. To ensure that differences in neural dynamics between the groups would not derive from possible difficulties in processing variable phonology in the dyslexics^32–34^ and to make the procedure minimally challenging for the young participants, only a single novel word with a simple phonemic structure was extensively repeated. Furthermore, to specifically target the phonological aspects of acquisition, we attached no meaning to the novel spoken stimulus, as semantics might even compensate some of the learning difficulty in dyslexia^8^. Dyslexic and normal-reading control children were briefly (11 min) exposed to a repetitive novel spoken word-form. We analysed the pattern, speed, and differences of exposure-related neural response dynamics to the novel item between the two groups, as well as the relationship with reading and writing performance. We hypothesised that fast learning-related brain response increase would be observed in children with normal reading ability^31^. Furthermore, we hypothesised that since dyslexic children show slower word learning behaviourally^9,10,12^, this would be reflected in reduced neural learning dynamics in brief exposure to the purely phonological novel word-form. Finally, akin to the dysfunctional neural suppression to stimulus repetition in dyslexia^23^, we hypothesised that dyslexics would not benefit from increased repetition in the memory-trace build-up to a novel verbal stimulus, shown as impaired neural dynamics to extensive repetition.

## Materials and Methods

### Subjects

Forty-five right-handed 9–12-year-old native Finnish-speaking subjects were recruited from schools in the Helsinki metropolitan area. They were screened with a battery of neuropsychological tests (see Neuropsychological measures for full description) to determine if they met inclusion criteria of either the dyslexic or the control group. One of the subjects in the dyslexic group was excluded due to a history of delayed speech onset and a suspicion of speech production impairment; one was excluded for scoring below two standard deviations (SD) of the normative mean (i.e. below average) in the verbal reasoning measure; and one for excessive movement artefacts in the EEG data. In the control group, one subject was excluded due to performance above two SD (upper extreme) of the normative mean in the perceptual reasoning as well as working memory indices; and one for excessive alpha rhythm in the EEG. This resulted in matched groups of 20 children with dyslexia (mean age 11.17 yrs (SD = 1.08), 11 male) and 20 normal reading controls (mean age 10.76 yrs (SD = 0.95), 10 male). All subjects had normal (>85) non-verbal IQ. The children gave an oral informed assent and their parents a written informed consent to participate in the study, which was approved by the Ethical Review Board in the Humanities and Social and Behavioural Sciences of the University of Helsinki and all procedures were conducted in accordance with the Declaration of Helsinki.

All included subjects attended a normal school, were reported to have normal hearing as well as normal or corrected-to-normal vision, and no neurological or neuropsychiatric disorders. In the dyslexia group, one subject had received neuropsychological rehabilitation and 18 had received special tuition in school for their reading difficulties. Eleven children in this group had been formerly tested for having dyslexia by either a psychologist or a special education teacher. Ten children without an official statement of dyslexia had a history of difficulties in learning to read and sustained reading difficulties. A licensed psychologist confirmed their dyslexia with the neuropsychological tests used in the current study, with inclusion criteria of scoring in the 10^th^ percentile of the age-normative scale in the reading task, or ≤20^th^ percentile in both the reading and writing tasks. Two of the children with an earlier dyslexia status had established compensatory strategies and scored above normative 20^th^ percentile in the reading task, which did not lead to exclusion. The rest of the children with an earlier assessment fulfilled the criteria for dyslexia described above.

### Neuropsychological measures

Subjects’ non-verbal and verbal reasoning, verbal memory, rapid naming, and reading and writing skills were assessed in a single session on a separate day before the EEG experiment. Perceptual non-verbal reasoning was assessed with the Perceptual Reasoning Index (PRI; subtests Block Design, Matrix Reasoning, and Picture Concepts) of the Finnish version of Wechsler Intelligence Scale for Children IV (WISC-IV^35^). Similarities subtest of WISC-IV was used as the verbal reasoning measure. Phonemic awareness was assessed with the Phonological Processing subtest of NEPSY-II (A Developmental Neuropsychological Assessment^36^). Verbal short-term and working memory was assessed with the Working Memory Index of WISC-IV (WMI; subtests Digit Span and Letter-Number Sequencing) and Word List Interference subtest of NEPSY-II. Visual-verbal associative learning and delayed retrieval was assessed with Memory for Names of NEPSY-II. Rapid automatized naming (RAN) was tested with subtests Colours, Letters, Numbers, and Objects, and rapid automatized switching (RAS) with Letters-Numbers and Colours-Letters-Numbers subtests from the Rapid Automatized Naming test^37,38^. Reading fluency (correctly read words in 2 min from a list of words of increasing length and difficulty) and writing accuracy (spelling words and sentences of increasing length and difficulty from dictation) were assessed using Lukilasse test^39^.

### Stimulation and procedure

The stimulus was a spoken tri-syllabic CVCVCV Finnish pseudo-word ‘tatata’, constructed by cross-splicing three naturally spoken syllables. Previous studies have found abnormalities in the perception of within- and between-category phoneme and syllable contrasts in developmental dyslexia^32–34^. Henceforth, we used acoustically identical syllables to create the tri-syllabic novel word-form, so that possible differences in processing the acoustic-phonetic features of the syllables between the dyslexic and control groups could not explain their responses to the spoken stimulus.

This word-form diverges from existing words early, at the beginning of the second syllable. Words beginning with ‘tat-’ in the Finnish lexicon are few and rare: 19 words (Dictionary of Standard Finnish, www.kielitoimistonsanakirja.fi) with a sum frequency of occurrence of 0.6 instances per million/log transformed −0.22 (Corpus of Finnish Magazines and Newspapers from the 1990s and 2000s, https://korp.csc.fi/download/lehdet90-00). Importantly, the word-form conforms to Finnish phonological structure and resembles real words (e.g. ‘tavata’ = to meet, ‘kokoko’ = interrogative form of ‘the size’) despite the reiteration of a single syllable. We used a syllable ‘ta’ of 100 ms in duration, uttered by a native female speaker to a high-quality condenser microphone (C4000B, AKG, Vienna, Austria), recorded with an analogue-to-digital converter (Digidesign DIGI-002, Avid Technology, Inc., Burlington, MA, USA) in a sound-attenuated booth. The loudness of the syllable was first normalized by the maximum peak amplitude after which it was reduced by 6 dB. The pitch was 169.3 Hz and voice onset time 12 ms. In order to have all the syllables of the pseudo-word acoustically identical, the produced syllable was then cross-spliced three times in succession with 50 ms silent inter-syllable gaps, the stimulus duration thus being 400 ms. Six filler stimuli in which the middle or final syllable differed from the frequent stimulus by vowel, pitch, or duration (none of which made the stimulus a real word), were additionally prepared. Praat^40^ and Adobe Audition 3.0 (Adobe Systems Inc., San Jose, CA, USA) software were used for stimulus preparation.

The subjects were seated in an acoustically and electrically shielded EEG chamber and instructed to ignore the presented spoken stimuli, delivered at 55 dBA SPL through headphones, and to watch a silent self-selected film without subtitles. To reduce habituation arising from repetitive presentation 30% of trials were pseudo-randomly occurring filler items, while the novel word-form constituted 70% of the stimulus sequence (ERP dynamics to the filler items were not analysed due to insufficient signal-to-noise ratio). Stimuli were presented in three fixed sequences, and their presentation order was counterbalanced across groups. The mean stimulus onset asynchrony (SOA) was 800 ms, jittered in 10-ms steps within the range of 750 to 850 ms. In total, the novel word-form was repeated 540 times during an 11-minute session.

### EEG recording and pre-processing

The EEG was recorded at a 512 Hz sampling rate with a DC-104 Hz bandwidth using an active electrode Biosemi ActiveTwo system (Biosemi B.V., Amsterdam, Netherlands) with 64 channels, referenced to the PO1 electrode site. Further electrodes were placed on the mastoids, and below and at the temple close to the corner of the right eye for the recording of eye movements (EOG). Data were offline downsampled to 256 Hz, noisy channels were interpolated, and principal component analysis (PCA^41^) was used to remove components caused by eye blinks or saccades. Further, a 1–30 Hz filter was applied and the data were extracted to epochs of −100–900 ms in relation to the stimulus onset. Epochs containing artefacts exceeding a threshold of ±100 µV were discarded from further analysis. After artefact rejection, the average total number of epochs was 455 (SD = 49) in the control and 465 (SD = 57) in the dyslexic group (difference n.s.: t(38) = 0.58, p > 0.5). Data were referenced to the average of mastoid signals and baseline was corrected on the 50 ms silent interval before the second syllable onset, i.e. the point in time when the stimulus disambiguates from other possible words in the native lexicon. To avoid inclusion of neural activity deriving from possible transient attention-shift to the spoken stimuli, first five trials were excluded from further analysis. Data from the beginning to end of the session were divided in four blocks, and epochs in each were averaged together. This guaranteed a sufficient SNR in each block, with 135 trials presented during 2.75 minutes per block.

In order to model the neural source activity corresponding to any learning-related neural dynamics at the sensor level, their underlying cortical generators were estimated using weighted minimum-norm current estimate (wMNE^42^). The minimum-norm method localises cortical neural activity from sensor-recordings by estimating the combination of active neural currents with a model of the surface voltage distribution with minimal total activity^43^. Signal from all sensors were included in the source reconstruction. First, age-appropriate MRI templates with 6-month intervals (obtained from Neurodevelopmental MRI Database^44^) were segmented with Brainsuite software^45^ using the BCI-DNI brain atlas^46^. These segmented templates were then utilised to create boundary element models (BEM) in Brainstorm software^47^ with 1922 vertices per layer (scalp, and inner and outer skull with 4 mm thickness). As the wMNE source reconstruction was applied on EEG data, unconstrained dipole orientations were chosen. Finally, for the purpose of statistical analysis, source activations were projected onto an average 10.5-year-old template (Neurodevelopmental MRI Database^44^).

### Statistical analysis

Performance in the neuropsychological tests was quantified by multivariate ANOVA using normative standard scores for the WISC-IV and NEPSY-II variables. A separate MANCOVA was calculated for reading, writing, RAN, and RAS, for which raw scores were used with age as a covariate.

**Figure 1:**
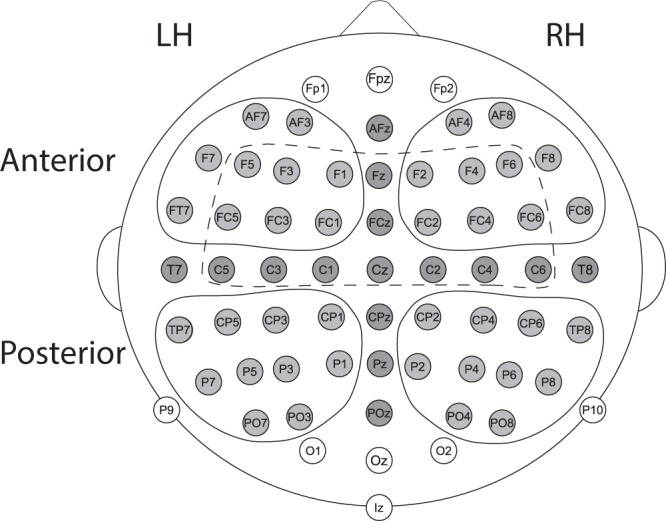
Regions-of-interest (ROIs) in ERP analysis. Channels inside the dashed line indicate the ROI used for peak latency detection. Channels highlighted in grey comprise the ROI that was used in the main ERP analysis. Following the main analysis, the topography of significant effects was examined with four ROIs in the left (LH), right (RH), anterior, and posterior plane (outlined with solid lines).

Event-related potentials were quantified relative to the divergence point (DP) at the second syllable onset. Focus in the analysis was set a priori on the first negative response as to its pertinence to word memory-trace formation established in previous studies^28–31^. Peak latencies were extracted from a fronto-central region-of-interest (ROI; Fig. 1) in which the group average responses were strongest for all investigated deflections. Peak latencies were individually determined for each subject by defining the first negative peak after DP from the individual average ERP. Later responses were determined as the two consecutive deflections. Group differences between peak latencies were tested with a t-test.

Mean amplitudes were extracted from 20 ms time windows around the individual peaks from a ROI covering the topographical spread of the response on the anterior-to-posterior and left-to-right hemispheric plane (see Fig. 1). Specific interest in the exposure-related neural dynamics was on the response change across the number of repetitions that was earlier shown to elicit a significant response increase for novel word-forms, i.e. 150–160 trials^28–30^, which corresponded with the first half of the exposure. However, since dyslexics were hypothesised to demonstrate slower learning, we wanted to test whether they showed response change later on, i.e. between the initial and final halves of the exposure, or even within the final half. Therefore, mean amplitudes from each group and block were submitted to a repeated-measures analysis of variance (rmANOVA) with factors Group (dyslexic vs. control) × Block (initial vs. final half of exposure) × Sub-block (first vs. second in each block). Responses with significant changes were followed up with a topographical analysis, which comprised ROIs in anterior and posterior sites in the left and right hemispheres (Fig. 1). The amplitudes were submitted to rmANOVA with factors Group × Block × Sub-block × Hemisphere (left vs. right) × Frontality (anterior vs. posterior ROI). Subsequent responses were analysed ad hoc similarly to the first response. Bonferroni-correction was employed for multiple comparisons.

Source-space analysis was performed in accordance with the sensor space analysis, by which the mean current densities were extracted from the same individual time windows. In order to localise the underlying sources for the enhancement in sensor-space, we ran planned comparisons of the mean current densities of the pertinent blocks with one-tailed t-tests for each vertex of the boundary element model.

Finally, associations between reading, writing and significant sensor-level exposure-related response changes across and within groups were investigated. All possible correlations were analysed since it could not be ascertained a priori whether performance in literacy measures and rapid neural learning stem from similar mechanisms in the two groups. Standard scores of the Reading fluency and Writing accuracy tests were correlated with significant exposure-related neural response change. Group differences between significant coefficients were analysed using univariate ANOVA by comparing the effect of scores on the response change. Statistical analyses were carried out with SPSS Statistics 23 software (IBM Corp., Armonk, NY, USA).

## Results

### Neuropsychological assessment

Verbal neuropsychological tests indicated typical developmental dyslexia deficits^36,38,48,49^ in our reading-impaired subjects (Table 1). There was a significant effect of group both in the MANOVA for WISC-IV and NEPSY-II tests (F(13,26) = 2.9, p < 0.01) as well as the MANCOVA for RAN, RAS, and literacy measures (F(6,32) = 14.61, p < 0.001). Dyslexics performed significantly worse than controls in tasks of reading fluency and writing accuracy, phonological processing, paired-associate delayed recall, and verbal reasoning (notably, however, verbal reasoning was within the normative average), and were slower in rapid naming and switching. The groups did not differ significantly in their perceptual reasoning abilities.

**Table 1:** Neuropsychological assessment.

Measure	Control (n = 20)	Dyslexic (n = 20)	Group difference			
			d	F	p-value	
PRI	111.65 ± 10.78	108.7 ± 11.64	0.263	0.692	0.411	
Block Design	11.8 ± 2.48	10.95 ± 3.05	0.306	0.933	0.340	
Picture Concepts	11.75 ± 2.55	11.75 ± 2.4	0.0	<0.001	1.000	
Matrix Reasoning	11.7 ± 2.45	11.25 ± 2.61	0.178	0.367	0.549	
Similarities	12.15 ± 1.79	9.5 ± 2.82	1.122	16.725	<0.001	*
WMI	104.95 ± 11.15	96.25 ± 11.59	0.765	5.85	0.02	
Digit Span	10.2 ± 2.75	9.4 ± 2.52	0.303	0.921	0.343	
Letter-Number Sequencing	11.35 ± 1.87	9.35 ± 2.5	0.906	8.212	0.007	
Word List Interference	9.75 ± 2.51	8.35 ± 2.39	0.571	3.262	0.079	
Memory of Names	10.65 ± 2.81	8.4 ± 2.09	0.909	8.244	0.007	
Immediate	10.45 ± 2.98	8.6 ± 2.11	0.717	5.125	0.029	
Delayed	11.15 ± 2.3	8.35 ± 2.25	1.231	11.154	0.002	*
Phonological Processing	12 ± 1.75	8.15 ± 3.08	1.537	23.612	<0.001	*
RAN errors^†^	5.3 ± 4.08	6.6 ± 4.35	0.308	1.467	0.233	
RAN speed (s)^†^	141.4 ± 19.27	163.0 ± 18.81	1.134	17.208	<0.001	*
RAS errors^‡^	2.3 ± 2.15	3.9 ± 2.15	0.744	7.557	0.009	
RAS speed (s)^‡^	68.3 ± 10.99	84.2 ± 11.94	1.386	25.733	<0.001	*
Reading Fluency						
raw score	97.95 ± 8.68	66.10 ± 19.12	2.145	74.518	<0.001	*
standard score	12 ± 1.34	5.05 ± 2.96				
Writing Accuracy						
raw score	41.45 ± 7.31	35.75 ± 11.34	0.597	9.795	0.003	*
standard score	11.35 ± 1.81	6.55 ± 3.46				
Age (years)	10.76 ± 0.95	11.17 ± 1.08	0.403	1.271_(t)_	0.211	

Mean ± standard deviation are shown. Group comparisons of normative standard scores (mean = 10 ± 3 for subtests and 100 ± 15 for scales), effect size (Cohen’s d), F-statistic and p-values obtained with a MANOVA for the subtests of WISC-IV, comprising Perceptual Reasoning Index (PRI) and Working Memory Index (WMI), verbal reasoning (Similarities) and NEPSY-II (Word List Interference, Memory For Names and Phonological Processing). For Rapid Automatized Naming and Switching (RAN and RAS, respectively), Reading Fluency (correctly read words in 2 min), and Writing Accuracy (words and sentences from dictation), raw scores were compared in a MANCOVA with age as covariate. Age distribution between groups was tested with a two-tailed t-test. P-values with Bonferroni correction at α = 0.05/20 = 0.0025 are denoted with an asterisk (*).

^†^Composite of 4 subtests (Colours, Numbers, Letters, Objects).

^‡^Composite of 2 subtests (Letters-Numbers, Colours-Numbers-Letters).

### Early exposure-related neural dynamics

The first peak after the DP was analysed for possible exposure-related dynamics (see Methods for details). In control children, the response amplitude exhibited an increase due to repetition of the novel word-form within the first 6 minutes of exposure, coherently with a study on Danish speaking 5–12-year-old typically developing children^31^. Crucially, this rapid response increase, thought to indicate automatic neural memory-trace formation for the new word and shown to predict later memory for the words^30^, was not established in the dyslexic children during the whole period of the 11-minute exposure.

**Figure 2:**
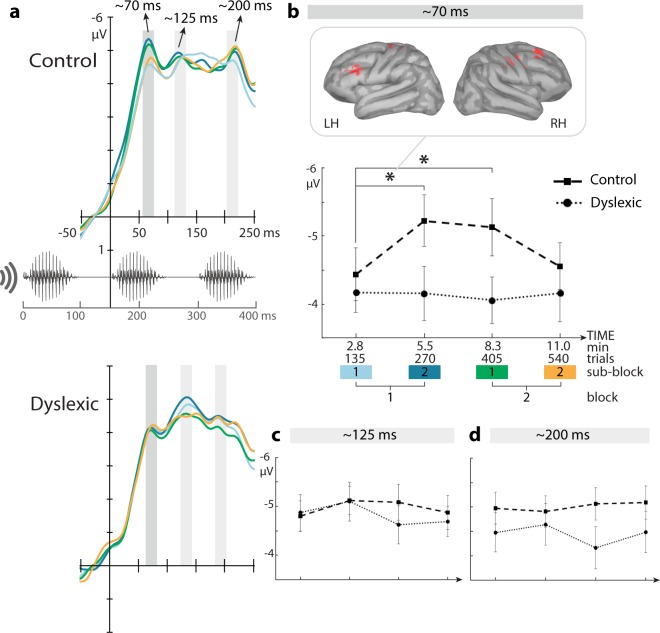
ERP responses to the novel word-form across the exposure. (**a**) Three negative-going responses (highlighted with grey bars) occurred after the critical second syllable onset, i.e. the word divergence point indicated by the y-axis. The sound waveform shows the temporal co-occurrence of the speech stimulus. ERP curves (**a**) and line graphs (**b**) of mean responses per sub-block over the course of the 11-minute exposure depict a significant increase of the early response at ~70 ms within the first block in the controls (squares and dashed line), whereas no change in amplitude was established in the dyslexic group (circles and dotted line) during the entire exposure period. The cortical origins of the response increase are shown in the source activity map in red (p < 0.025, uncorrected). The later responses at ~125 ms (**c**) and ~200 ms (**d**) did not show significant response changes in either group. Error bars denote SEM. *p < 0.05.

**Figure 3:**
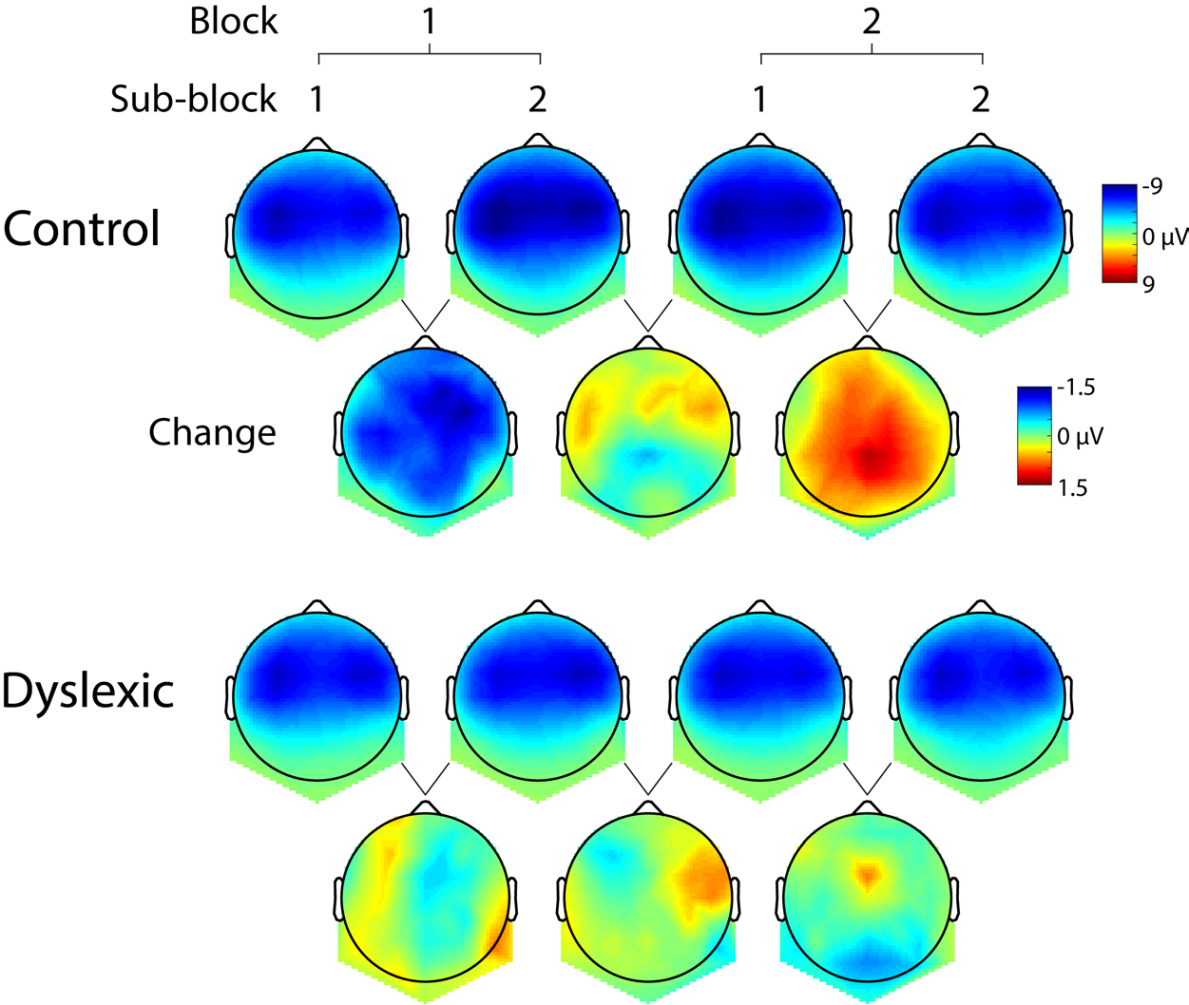
Scalp topographies and signal change between blocks for the early response in the control and dyslexic groups. Topographies for each block and sub-block, and signal change between consecutive blocks of mean amplitudes in a 20 ms time window around individually defined peaks.

To quantify this electrophysiological dynamics, and taking into account the generally considerable variance in ERPs of children (e.g. Bonte *et al*.^50^; for review, see Ponton *et al*.^51^), the response latencies were first acquired for each subject individually. The average latency of the first response was significantly different between groups: 65 ms (SEM = 1.85) in the controls and 73 ms (SEM = 2.26) in the dyslexics (t(38) = 2.55, p = 0.015). This indicates a delay in the neural response to the stimulus disambiguation point (second syllable onset) in the dyslexic subjects. Critically, the response amplitude dynamics were also significantly discrepant between the two groups in the initial and final half of the exposure (Fig. 2a response at ~70 ms). Interaction Group × Block × Sub-block was significant (F(1,38) = 4.29, p = 0.045). Post hoc pairwise comparisons showed that in the control group the response significantly increased in negativity in the initial half of the session (i.e. between the first and second sub-blocks, p = 0.017), the difference lasting until the first sub-block of the final half (p = 0.047; Fig. 2b). In contrast, in the dyslexic group, no change during the entire exposure occurred (all p-values > 0.75). The significant interaction between the groups corresponded with a significant quadratic contrast between the groups; i.e. the linear dynamics of controls’ responses through the entire exposure fitted a quadratic function (p = 0.045). The investigation of the response topography in both groups (Fig. 3) followed the main analysis, by which the response was generally more prominent in the anterior than posterior locations (F(1,38) = 283.39, p < 0.001), and these anterior responses were stronger in the right than left hemisphere (F(1,38) = 5.32, p = 0.027).

### Late ERP dynamics

Following the early peak, two further deflections were observed (Fig. 2a responses at ~125 ms and ~200 ms). The mean latencies of the second response, 124 ms (SEM = 3.15) in the control and 131 ms (SEM = 2.99) in the dyslexic group, did not significantly differ between the groups (t(38) = 1.62, p = 0.114). No significant interactions or main effects of group or blocks/sub-blocks were found in the analysis of the mean response amplitudes (F(1,38) < 1.2, p > 0.3). For response topographies, see Supplementary Fig. S1.

The third peak occurred at 206 ms (SEM = 4.99) in the control and 193 ms (SEM = 4.257) in the dyslexic group, with a non-significant group difference (t(38) = −1.20, p = 0.053). These latencies corresponded to 56 and 43 ms after the final syllable onset, possibly reflecting a reaction to this syllable. Similar to the second deflection, the response amplitudes demonstrated no significant interactions or main effects (F(1,38) < 1.66, p > 0.2). For response topographies, see Supplementary Fig. S2.

In order to verify that the significant exposure-related dynamics to the first response following second syllable onset was not driven by earlier effects or merely related to single-syllable processing per se, we repeated the analysis on the responses established to the initial and final syllables, with baseline corrections set to 50 ms intervals before each syllable similarly to the main analysis (see Supplementary Information). If the significant increase found for the lexically critical response was related to processing of the specific syllable ‘ta’, similar increase should be discovered for each syllable of the word-form. This, however, was not the case. Responses to the initial or final syllable did not show any effects indicating response changes to exposure (see Supplementary Information). P1 response was generally larger in amplitude in the control than dyslexic group, akin to findings in a previous child study using speech stimuli^50^. Furthermore, there was a main effect of sub-block, by which the first sub-blocks were smaller in amplitude than the second sub-blocks. Since such effect occurred across the two blocks, irrespective of the accumulation of exposure from the first half (or block) to the second, this pattern cannot be considered an indication of change due to increasing exposure. While the P1 result verified that our main finding of neural learning was not established for this early obligatory response to a sound stimulus, the analysis of the response to the final syllable with a baseline correction set prior to the syllable onset validated that such enhancement was not present for the consecutive syllables in general. Indeed, it seems the response increase was selectively related to the disambiguation time point that was critical for the identification of the unfolding speech stimulus to be a novel word, i.e. a word-like item that had not previously had a long-term memory trace in the neural lexicon.

### Source dynamics

Significant exposure-related dynamics at sensor level were followed up by a distributed source analysis of the underlying cortical generators. For the sensor-level increase within the first block in controls, paired one-tail t-tests with α < 0.025 indicated corresponding source activation enhancement in bilateral frontal cortices (Fig. 2b). More specifically, the sources were localised in the left inferior frontal gyrus (MNI coordinates x = −43, y = 25, z = 23), bilateral precentral gyrus (-24, -8, -72 and 43, -11, 49), bilateral postcentral gyrus (-17, -27, 64 and 60, -15, 42), right middle frontal gyrus (46, 7, 42 and 36, 34, 39) as well as right superior frontal gyrus (23, 32, 47). No significant increase in source activations was observed in the dyslexic group. Supplementary Fig. S3 shows the original source activations in the first and second sub-blocks of the first block in both groups.

### Associations of reading and writing skills with learning-related neural dynamics

**Figure 4:**
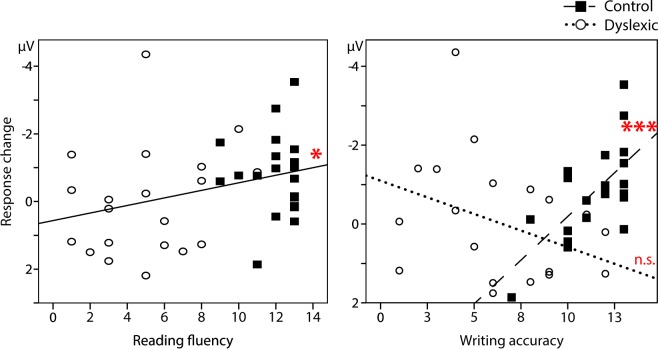
The relationship between literacy scores and the neural response change. With merged groups (squares representing controls and circles dyslexics), larger negative-going response increase was associated with better reading fluency (r = −0.321, p = 0.043). Similar association was found with writing accuracy, but only in the control group (r = −0.667, p = 0.001). Note that some subjects in the dyslexic group, who had been previously diagnosed with dyslexia, had established compensatory strategies in reading, performing in the normative average range. In the literacy measures, normative standard scores are presented. Asterisks indicate coefficient significance, *p < 0.05, ***≤0.001.

To examine whether the learning-related neural dynamics occurring during the first 6 minutes of exposure was associated with literacy skills, we correlated the standard scores of Reading fluency and Writing accuracy across and within groups with the early ERP response increase (Fig. 4). Pearson’s correlation showed that when groups were combined, there was a significant correlation with Reading fluency (r = −0.321, p = 0.043), indicating larger response increase with increasing reading fluency. This was not significant when groups were correlated separately (r < −0.24, p > 0.3). On the contrary, Writing accuracy correlated with the neural response change such that only within the control group stronger response increase was associated with better writing (r = −0.667, p = 0.001). Writing accuracy showed no significant correlation in the dyslexics (r = 0.364, p = 0.114) nor across groups (r = −0.135, p = 0.407). Univariate ANOVA with the response change as the dependent variable showed a significant interaction of Group × Writing accuracy (F(1, 36) = 11.49 p = 0.002) which verified that the correlation coefficients between the groups differed significantly.

## Discussion

We neurophysiologically assessed online changes in neural dynamics elicited by a novel word-form in matched groups of dyslexic and normal-reading control children during passive perceptual exposure. The control children demonstrated a significant increase in the early neural response at 65 ms after the word divergence point, that is, when sound input allowed for lexical disambiguation. This response enhancement occurred remarkably fast within the first 6 minutes of repetitive exposure to the novel word. This finding aligns with previous results of robust neural response enhancement reflecting automatic cortical memory-trace build-up of novel word-forms (confirmed behaviourally) in adults and children^28–31^. Here, however, we showed that such response enhancement was absent in the dyslexic children.

Strikingly, even doubling the exposure duration and the number of repetitions (in comparison to the time the response increase was established in controls) was not sufficient to elicit the exposure-related neural enhancement in dyslexics. This indicates an inability of the dyslexic brain to quickly change its response dynamics, and thus suggests a deficit in the memory-trace development for new word-forms through perceptual exposure. The results are in line with the anchoring deficit^15,16^ and impaired Hebbian learning accounts in dyslexia^52^; our dyslexic participants did not show the type of sensitivity to repetition exhibited by the controls. Possibly, the neural perceptual system of the dyslexics may have treated the repeated word-form each time as a novel one, not showing a learning effect. Neurophysiologically, the usually expected effect of repetitive stimulus presentation is response attenuation; passive ERP-paradigms similar to the one employed here have indeed shown such a decrease for both known words and non-speech stimuli^27–29,53,54^. Thus, the response increase for novel word-forms in fact counteracts and overrides any suppression effects^30^. Furthermore, while it was previously found that dyslexic children and adults show reduced neural adaptation to repeated familiar spoken words compared to controls^23^, it has remained unknown whether the abnormal neural suppression may correspond to a similar defect in neural enhancement. This latter question is answered by the current study which demonstrates the absence of response enhancement to novel unfamiliar spoken words during perceptual exposure in dyslexic children. Together with the reduced adaptation finding of Perrachione *et al*.^23^, these results provide novel insights into the neurophysiological learning mechanism in dyslexia in that both rapid suppression and enhancement are impaired, possibly due to dysfunction of the neuronal resources shared by these mechanisms. Essentially, in dyslexic individuals the mechanism shows ‘resistance’ to the beneficial effects of repetition in the memory-trace foundation, be it a short-term sensory one or the initial stage of encoding a novel item into the long-term memory.

The latency of the individually defined peaks of the early response differed significantly between groups, occurring at 65 ms in the control and 73 ms in the dyslexic group. This is a slight yet sizeable (>10%) latency increase, suggesting a delay in the stimulus processing. It is important to note that while this early response to the second syllable could be interpreted as a late response to the first syllable, the unfolding of the acoustic-phonetic input of the second syllable interferes late processing of the first syllable. Critically, the linguistically crucial activity dynamics diverges after this disambiguation point, endorsing the interpretation of the response reflecting processing related to the second syllable. Previous reports have shown that the first event-related response peaking at 50–100 ms after the sensory information becomes sufficient to identify lexical status indicates automatic memory circuit activation for words and lack of activation for pseudo-words^55,56^. The early latency of the current response conforms to these findings. The delay in the dyslexics is in line with anomalous processing of early phonological cues in spoken word recognition^50,57–59^, further emphasising the observed discrepancy between groups in the memory encoding of the novel item. While the latency increase is small, it is nevertheless significant, and its duration (~8 ms) exceeds that of a single synaptic transmission, an important step in neural information processing, and this result therefore may indicate a delay in functional connectivity and information transfer in the brain^60,61^.

The source reconstruction localised the origins of the cortical response enhancement in the control children to the posterior LIFG (BA44), right dorsolateral prefrontal cortex (DLPFC BA8 and 9), as well as in bilateral pre- and post-central gyri (BA6 and BA4, respectively). We found no significant increase in source activations in the dyslexic group. The increase in source activity was partially similar to that seen in healthy adults, who recruit the LIFG and temporal cortex in the rapid word-form memory build-up^28–30^, suggesting a prominent role of LIFG in the rapid automatic word learning. Increased right DLPFC (BA9) activation, on the other hand, was found to pertain to enhanced confidence in correct recognition of words as previously encountered^62^ and familiarity effects in recognition memory more generally^63^. These latter findings could help explain our results, possibly reflecting increased familiarity with the repeated word-form. At the same time, in an MEG study of 5–12-year-old Danish children, Partanen *et al*.^31^ reported no LIFG or DLPFC sources involved in the response increase to novel native language word-form. However, they did not include DLPFC in their source analysis ROIs; more generally, MEG is less sensitive than EEG in detecting frontal sources^64^, especially at the crest and troughs of sulci^65^. While the shortcoming of EEG source reconstruction is the lack of high spatial precision and the exact source locations in the current study without individual MRIs (which were avoided considering the age of our subjects) should be taken with caution, the results point to frontal involvement in the word memory-trace development during brief exposure. The absence of the frontally-generated response enhancement in the dyslexics could refer to abnormalities in LIFG and DLPFC function in dyslexia. Akin to our results, dyslexic children were previously found to recruit LIFG and bilateral MFG less than controls during difficult tasks of phonological and lexical processing^66–69^. Furthermore, dyslexic children have shown reduced neural adaptation to spoken familiar words in LIFG, in addition to temporal locations^23^. Moreover, reduced functional connectivity between the left posterior temporal areas and the LIFG was consistently found during reading, lexical decision, and resting state in dyslexic individuals^70^. Potentially, this reduced connectivity may also explain the latency delay discussed above. Our results thus suggest that the automatic neural learning dysfunction with regard to novel word repetition in dyslexia might be underpinned, at least in part, by the lack of frontal engagement.

Critically, larger neural enhancement was associated with better reading fluency across groups. This corroborates the initial finding of a group difference but additionally elucidates the nature of the relationship of the neural learning effect and reading fluency irrespective of categorical group designation. Namely, the individual response change magnitudes spread into a linear continuum associated with reading performance, indicating that rapid neural memory-trace formation ability accounted for variability in reading. Previously, reading skills were shown to correlate with the degree of preserved adaptation to spoken familiar words in dyslexic adults and children^23^. Moreover, writing accuracy had a similar association with the enhancement but only in the controls. The lack of this association across groups or within dyslexics may reflect a lack in using shared neural resources in the writing task and neural learning from repetition. Namely, as accurate spelling in controls may be a fast, automatized process, it possibly requires further cognitive effort in dyslexics. Here, as the writing task did not have a time limit and self-correction of initial mistakes was allowed, the outcome in dyslexics conceivably reflects additional processes compared to controls. It thus seems that reading and to some extent writing, which are often both affected in dyslexia, have a relation to rapid neural learning dynamics. The function of this neural learning mechanism may underlie the acquisition of phoneme-to-orthography mappings, which is a pre-requisite for reading and compromised in dyslexia (for review, see Vellutino *et al*.^4^).

In addition to the early response, two later peaks followed. The latency of the first one at 124 ms in controls and 131 ms in dyslexics (difference n.s.) supposedly reflected secondary processing of the lexically critical second syllable. This response manifested no significant changes in amplitude during the exposure or between groups. In adults, a similar second-order response to known and novel word-forms during perceptual exposure showed effects of attention manipulation^30^, suggesting that this response may be linked to the amount of attention that is allocated to the speech stimuli. The current paradigm employed only a passive listening condition in which subjects were instructed to ignore the stimuli and concentrate on a silent film. Thus, assuming this later response is attention-related, the current result of no exposure or group effects would suggest that subjects in both groups were focusing their attention away from the speech stimuli to similar extent and that marked fluctuations of attention did not play a crucial role here. The short exposure time of 11 min in total and the absence of a cognitively demanding task gives further reason to assume that attentional differences between groups were not considerable. The third response peak at 206 ms in controls and 193 ms in dyslexics (difference n.s.) occurred 56 and 43 ms post final syllable onset. Similar to the second response, there were no significant effects of group or exposure. This late response most probably indicated the processing of the final syllable and confirmative processing of the item. Such late responses have been hypothesised to result from second-order lexical search and re-analysis of language information^55,71^.

While the later responses did not show any significant exposure-related effects, we further tested if there were any effects relative to syllable onsets in general. This was done by replicating the analysis of the first response to the DP (second syllable), critically showing the neural learning effect, for the first peaks following both the initial and the final syllables. That is, baseline was corrected in an interval right prior to each of the syllables, and the first ERP response following the syllable onsets was analysed. None of these showed similar dynamics as the critical ERP deflection following the divergence point. These results confirmed that the significant response enhancement to the second syllable was not related to general phonological, phonemic or acoustic processing. This suggestion is further supported by the word-form make-up as all of the syllables being acoustically identical, the differential response dynamics to only one of them calls for an explanation. In this case, the co-occurrence of lexical disambiguation and the second syllable onset, as well as previous studies with similar response properties signifying word learning^30^, argue for the interpretation that the response enhancement reflects neural memory-trace formation for the novel word-form.

Crucially, our results pinpoint the deficit in the phonological aspect of early word-form encoding in dyslexic children in a paradigm without meaning acquisition or externally imposed semantic associations, articulation, or memory retrieval of the stimuli. This restriction was essential: although previous studies suggested the impairment of learning to be specific to phonological forms and not of any associations, they could not determine if the difficulties were in the encoding, retrieval and production of the right articulatory forms, or inferior verbal working memory in the learning phase^9,13,14^. Similar to these previous studies showing greater impairment in paired-associate learning of novel and less so of known words, our control subjects outperformed the dyslexic group in learning to associate familiar names to pictures of faces (as evident in the results of the ‘Memory for names’ test in our neuropsychological battery), even though the dyslexics’ performance was still within the normative average. Furthermore, we obtained the deficient neural dynamics in verbal learning in dyslexia using a perceptual-only passive exposure with minimal-to-none taxing of working memory or involvement of controlled attention. Thus, the results cannot be easily resolved by the account of a general short-term serial order memory deficit^72,73^.

As a word of caution, the exposure to a single novel word-form (as opposed to many) may be considered as a shortcoming of the current experimental paradigm, although this simple design was aimed at making it as effortless as possible for the young dyslexic participants. The extensive number of repetitions of a single item, interspersed with infrequently occurring fillers, in a short period of time, made it possible to ensure the children’s vigilance and attention on the silent film – critically decreasing the chance of attention-derived differences between the groups. This approach also allowed for investigating the possibility of dyslexics eliciting a response enhancement more slowly, with more exposure, by introducing a second block of stimuli, substantially exceeding the amount of repetitions used in earlier studies with healthy children and adults^28,30,31^. While this was not the case, the learning-related response in controls showed an observable tendency to decrease during the second half of exposure, which, however, was not statistically significant. This, in turn, provides confirmatory evidence of the number of repetitions needed to establish the response enhancement, and the stimulus becoming familiar, after which the response shows typical repetition-related suppression. Relatedly, one should acknowledge the limitations of the current experimental design when making interpretations of the results. Importantly, our aim was to study the neural mechanism underpinning the earliest stages of rapid automatic learning of word-forms. This design does not address the integration of newly learnt lexical forms into the existing mental lexicon, which remains to be tackled in future studies. Similarly, many behavioural word learning studies related to dyslexia did not test long-term memory of the new words^9,11,14^, leaving this question equally open. Furthermore, one should be cautious in interpreting the current results to account for ‘language learning’ in a general sense. However, since the neural pattern of responses in controls closely mimicked those obtained in the previous rapid word learning studies using neurophysiological measures, we have reasons to suggest that our results demonstrate impaired rapid word acquisition in dyslexia. Given the current study is the first to investigate the neural mechanisms of novel word learning in dyslexia, future studies need to confirm this initial finding.

Importantly, the results suggest that the phonological deficit in dyslexia indeed originates at the encoding of input^7,74^ and does not require output of the newly encoded phonological representation to be expressed, as some accounts have posited^14,75,76^. While the acquisition of new words may possibly be improved by attaching semantics or other associative information to the word-form in the learning phase, the inefficiency in word-form acquisition is proposed to result in phonologically less accurate and weak representations. Furthermore, the failure in establishing a neural memory trace to a single novel word within over 500 repetitions implies a serious phonological memory encoding deficit not just limited to slowness of learning. Indeed, a recent proposal of dysfunctional neuronal firing resulting in neural noise in dyslexia^77^ suggests that increased cortical excitability disrupts the synchronisation of neural activation to sensory stimuli in stimulus-specific cortical networks. Neural noise is suggested to lead to unstable phonological representations through poorly synchronised activity of different oscillatory bands that support the distinction of different phonemes and syllable segmentation^77^. The current result of deficient neural learning could be explained by this hypothesis, presuming noisy, inconsistent cortical activation by the stimulus hindering the response from being enhanced and any stable memory trace from being formed. Future research should test this account further by introducing different kinds of learning contexts and extending the investigation beyond the initial stages of learning to determine the scope of the deficit and possible compensatory mechanisms for the acquisition of new word representations in dyslexia.

## Electronic supplementary material

Supplementary information

## Data Availability

The datasets generated during and/or analysed during the current study are available from the corresponding author on reasonable request.
