# Supplementary material for: Impaired neural mechanism for online novel word acquisition in dyslexic children
Source: Sci Rep. 2018 Aug 24;8:12779. doi: 10.1038/s41598-018-31211-0 (PMC6109122; doi:10.1038/s41598-018-31211-0)
Supplement: Supplementary file 1 — Supplementary information [file 41598_2018_31211_MOESM1_ESM.pdf]

Impaired neural mechanism for online novel word acquisition in dyslexic children

Lilli Kimppa*, Yury Shtyrov, Eino Partanen, Teija Kujala

Supplementary Information

We analysed the responses following the initial and final syllable onsets, post hoc, with the same procedure as in the main analysis. Baseline correction was set to 50 ms interval before the onset of the pertinent syllable. Analyses were conducted on mean amplitudes that were extracted around individually determined peak latencies of the first responses following syllable onset. Identical with the main analysis, these mean amplitudes were tested with Group × Block × Sub-block repeated-measure ANOVA.

P1 to the initial syllable.

A positive response following stimulus onset, P1, occurred at 90 ms (SEM = 1.59) in the control and at 95 ms (SEM = 2.24) in the dyslexic group. This latency difference was not significat ($t(38) = 1.71$, $p = 0.095$). A significant main effect of group ($F(1,38) = 4.67$, $p = 0.037$) indicated stronger overall responses in the control (mean = 3.36 μV, SEM = 0.27) than the dyslexic group (mean = 2.71 μV, SEM = 0.31). Furthermore, there was a main effect of sub-block ($F(1,38) = 4.92$, $p = 0.033$), whereby average response amplitude was smaller for sub-block 1 (mean = 2.88 μV, SEM = 0.16) than for sub-block 2 (mean = 3.20, SEM = 0.18). Critically, however, there was no indication of linear change in either group, i.e. no significant main effect of block ($F(1,38) = 0.20$, $p > 0.65$) or interaction of group by block ($F(1,38) = 0.55$, $p >0.46$), block by subblock ($F(1,38) = 0.33$, $p > 0.57$) or group by block by subblock ($F(1,38) = 0.87$, $p > 0.35$).

Response to the final syllable.

The latencies of the response to final syllable were analysed in the main analysis. When correcting the baseline relative to the third syllable onset, we found no significant main effects or interactions ($F(1,38) > 1.81$, $p > 0.18$).

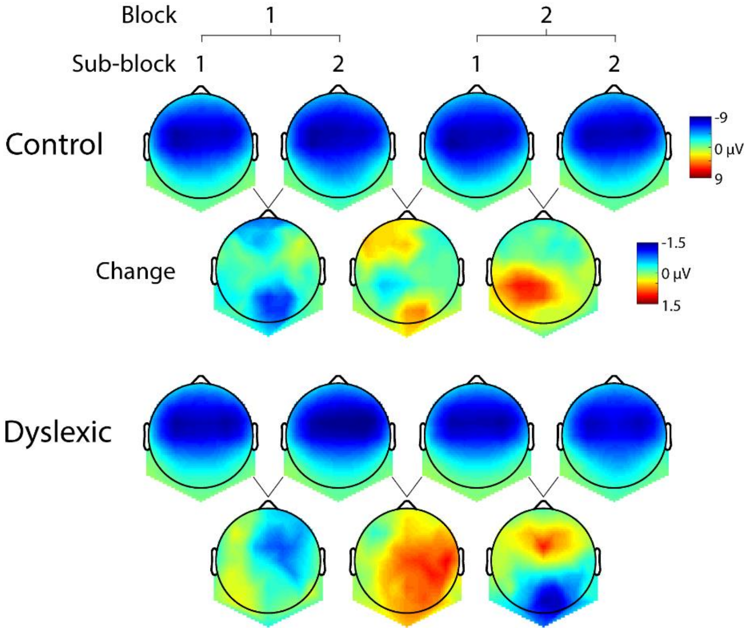

Supplementary Figure S1. Scalp topographies of the second response (mean amplitudes in a 20 ms time window around individual peaks), at 124 ms in controls and 131 ms in the dyslexic group, and topography changes between sub-blocks.

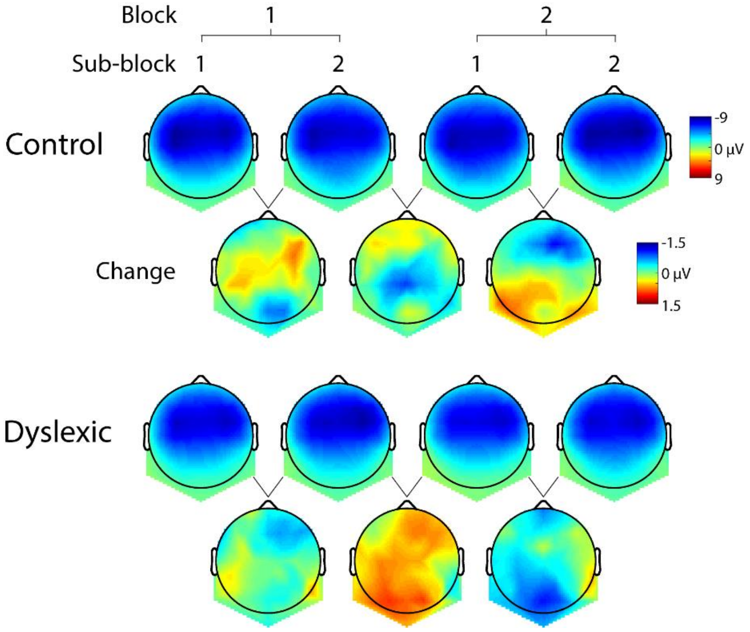

Supplementary Figure S2. Scalp topographies of the third response (mean amplitudes in a 20 ms time window around individual peaks), at 206 ms in controls and 193 ms in the dyslexic group, and topography changes between sub-blocks.

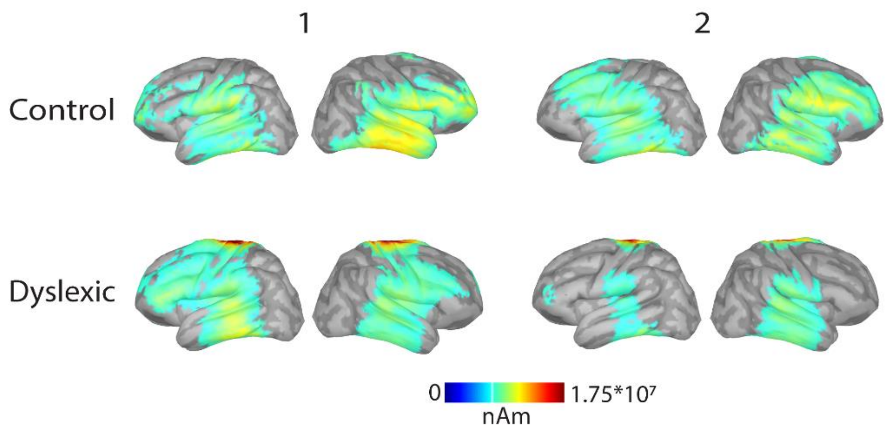

Supplementary Figure S3. Source activations in the first and second sub-blocks of the first block in both groups.
